# Comparison of Techniques and Solvents on the Antimicrobial and Antioxidant Potential of Extracts from *Acacia dealbata* and *Olea europaea*

**DOI:** 10.3390/antibiotics9020048

**Published:** 2020-01-28

**Authors:** Anabela Borges, Helena José, Vera Homem, Manuel Simões

**Affiliations:** LEPABE—Laboratory for Process Engineering, Environment, Biotechnology and Energy, Department of Chemical Engineering, Faculty of Engineering, University of Porto, 4200-465 Porto, Portugal; apborges@fe.up.pt (A.B.); bio08046@fe.up.pt (H.J.); vhomem@fe.up.pt (V.H.)

**Keywords:** *Acacia dealbata*, antibacterial resistance, antimicrobial activity, antioxidant capacity, extraction methods, *Olea europaea*

## Abstract

Ethnopharmacological use of plant natural extracts has been known since ancient times. The optimization of plant molecule extraction is fundamental in obtaining relevant extraction yields. The main purpose of this study was to understand the role of different extraction techniques (solid-liquid, ultrasound, Soxhlet, and microwave) and solvents (water, methanol, ethanol, acetone, dichloromethane, and hexane) on the antimicrobial and antioxidant activities of extracts from *Olea europaea* (olive) and *Acacia dealbata* (mimosa). Crude plant extracts were evaluated for their antimicrobial activity against *Staphylococcus aureus* and *Escherichia coli* by the disk diffusion method. The antioxidant capacity of the extracts was determined by ABTS (2,2-azinobis (3-ethyl-benzothiazoline-6-sulfonic acid)) and DPPH (2,2-diphenyl-1-picrylhydrazyl) methods. In terms of extraction yield, ultrasound extraction and the solvents methanol, acetone (*O. europaea*) or water (*A. dealbata*) were found to be the best options. However, ethanol and acetone proved to be the best solvents to extract compounds with antimicrobial activity and antioxidant capacity, respectively (regardless of the extraction method employed). Soxhlet and microwave were the best techniques to extract compounds with antimicrobial activity, whereas any of the tested techniques showed the ability to extract compounds with antioxidant capacity. In most of the cases, both plant extracts (mimosa and olive) were more efficient against *S. aureus* than *E. coli*. In the present study, both mimosa and olive leaf crude extracts proved to have antimicrobial and antioxidant activities, increasing the demand of these natural products as a source of compounds with health benefits.

## 1. Introduction

Since ancient times plants have been widely used as a sustainable source of biologically active products [1]. In fact, it is estimated that there are around 374,000 plant species in the world, and much more remain unidentified [2]. Plants produce a myriad of secondary metabolites known as phytochemicals. These are primarily used to protect themselves from external stresses and pathogen attacks [3]. Natural compounds from plant extracts are often used as source of food preservatives [4,5] and pharmaceutical agents [6,7] in the food and clinical sector, mainly with antimicrobial and antioxidant purposes. This is related to the growing demand for natural products to replace synthetic chemical compounds due to their associated adverse public health and environmental effects [4,5,8] and lack of activity [9]. Moreover, characteristics such as green and sustainable status, diversity of molecular structures, and distinctive/multi-target mode of action makes plant-derived products attractive for consumers [3,10]. The interest in using plants as functional foods or nutraceuticals is increasing, particularly taking into account new dietary habits, i.e., vegetarian and vegan diets. Therefore, numerous plants and/or physiologically active ingredients derived from plants have been explored for their nutritional health benefits, as well as in disease prevention and delay [11].

*Olea europaea* Linné (olive) is native to Mediterranean countries and is one of the oldest known cultivated trees in the world [12,13]. Olive fruits and olive oil are the two main olive tree products, constituting an important part of the Mediterranean diet. The regular consumption of these products has been associated with a low incidence of diseases (e.g., cancer and cardiovascular diseases) [12,14]. An enormous amount of by-products, such as crude olive cake, vegetation water, twigs, stems, and leaves results from the production of olives. Due to their rich content in bioactive compounds, they have also been considered as having valuable health effects and economic significance, which are still under-explored, thus gathering the interest of the scientific community and industries [15]. Among all by-products, olives leaves have attracted growing attention as they contain an array of simple phenolics (phenylethanoids, hydroxybenzoic acids, and hydroxycinnamic acids) and polyphenolic compounds (secoiridoids and flavonoids) with biological properties, including antioxidant and antimicrobial [16,17,18,19,20,21,22,23]. Currently, olive leaves extracts are very popular as nutraceuticals [24], as well as in infusions [25].

*Acacia dealbata* (mimosa) was originally found in Australia and Tasmania, but become invasive and in several parts of the world (Chile, Madagascar, South Africa, France, Spain, and Portugal) [26]. The biodiversity impact caused by *A. dealbata* has been significantly explored in America and Africa, but in Europe the knowledge on this subject is limited despite the dimension of the invaded areas [27]. An interesting aspect of this non-native plant is its high content in polysaccharides, which provides opportunity to fractionize the extraction of plant molecules by chemical methods to yield products suitable for different industries, namely, chemical, food, and pharmaceutical [28]. In fact, *Acacia* species are considered plants with economic importance as sources of tannins, gums, timber, fuel, and fodder, as well as of importance in traditional medicines. It is documented that biomass from this species (bark, flowers, leaves, pods, seeds, and roots) are rich in secondary metabolites (e.g., phenolics, flavonoids, terpenes, tannins, amines, alkaloids, and fatty oil) and have been used in traditional medicine for the treatment of various ailments (e.g., diabetes, worm infection, eczema, malaria, gout, jaundice, abdominal pain, kidney problems, constipation, leprosy, piles, pneumonia, rheumatism, fever, and cancer) [29,30,31,32].

In this study, leaves of *O. europaea* and *A. dealbata* were selected to assess the influence of the extraction technique (solid-liquid, ultrasound, Soxhlet and microwave) and the extraction solvents (water, methanol, ethanol, acetone, dichloromethane, and hexane) on the antimicrobial and antioxidant activities of their crude extracts.

## 2. Results and Discussion

In this work, the entire *O. europaea* and *A. dealbata* leaf extracts were selected for antimicrobial and antioxidant studies instead of their individual constituents. Actually, olive plants are rich in phenolic compounds for which their antimicrobial/antioxidant activities are well characterized [25]. Studies report that the antimicrobial and antioxidant efficiency of the olive leaf extracts are directly related with their content of polyphenols [18,20,33,34]. For instance, the activity (antimicrobial and antioxidant) of oleuropein, the most abundant constituent of olive leaves [35,36,37], has been extensibly studied and reported, but the available information is scarce on the entire extract [20,38]. Additionally, in general, in the mixed form, individual components can modify their properties and interact synergistically, producing improved effects [18,25,39]. Regarding mimosa leaves, information on the antimicrobial activity/antioxidant capacity of its extracts and individual active components is scarce [30].

### 2.1. Extraction Methods, Solvents, and Extraction Efficiency

Four extraction methods (solid-liquid, ultrasound, Soxhlet, and microwave) and six solvents (water, methanol, ethanol, acetone, dichloromethane, and hexane) were tested to determine the conditions for which extracts with higher antioxidant and antimicrobial activities were obtained. In the case of Soxhlet and microwave extraction methods, only water was used as extraction solvent, for safety reasons.

Table 1 shows the efficiency of all extraction methods and solvents. For *O. europaea*, the solid-liquid extraction efficiency ranged from 0.9% to 7.2%. In this extraction method, hexane was the weakest solvent and methanol the best one. Regarding *A. dealbata,* the efficiency ranged from 4.1% to 8.1%, where dichloromethane was the weakest solvent and water was the strongest (*p* < 0.05). No significant differences were found for the other solvents, for both plants (*p* > 0.05). According to Altıok et al. [35], the selection of the extraction solvent is the most important factor affecting the efficiency of solid-liquid extraction technique. These authors also found that mixtures of water/ethanol and water/acetone were generally more effective than the solvent alone in the extraction of polyphenolic compounds from *O. europaea* leaves.

For ultrasound extraction, dichloromethane (2.8%) and acetone (7.4%) were the solvents that showed the lowest and highest efficacy, respectively (*p* < 0.05). For the other solvents (hexane, ethanol, methanol, and water) the following efficiencies were obtained 3.2% < 4.6% < 6.0% < 6.2%, respectively. Shirzad and coworkers [40] studied the effect of different parameters (extraction time, temperature, ethanol-water ratio) in order to optimize ultrasound-assisted extraction of *O. europaea* leaves and concluded that ultrasound extraction is a method highly efficient, requiring shorter extraction time when compared with other methods. This is mainly related to mechanical effects (vibration/agitation and cavitation) that promotes the dissolution between immiscible phenol phases [41].

Soxhlet and microwave techniques using water as a solvent demonstrated good extraction efficiency at 12.3% and 10.7%, respectively. It has been described that microwave is an advantageous process of extraction, as it is simple, fast, and requires a small amount of solvent. Moreover, this technique facilitates the extraction of compounds with bioactivity due to the rapid heating of the matrix [21].

Concerning the mimosa leaves extracts, water (6.4%) also demonstrated higher extraction efficiency for the ultrasound method, whereas acetone (2.8%) showed lower efficiency (Table 1). A good extraction efficiency was also found with methanol as extraction solvent (*p* < 0.05) for both solid-liquid (7.9%) and ultrasound (5.7%) methods. In a study performed by Jerman et al. [42], methanol was considered the best solvent to extract phenols using the ultrasound method. This solvent did not degrade the phenols, as no hydrogen peroxide, neither large proportion of free radicals, are formed due to cavitation when exposed to sonication [43]. Ahmad et al. [44] reported that the different yields might be influenced by the polarities of the solvents, which can explain the variation in the results. Theses authors also showed that in the extraction of soybean isoflavones from *Elephantopus scaba* L., methanol was better extraction solvent than hexane, which is in agreement with the results of the present work for both olive and mimosa extracts. The study of Klejdus et al. [45] also corroborates these results. According to Cowan [46], methanol and water are the solvents that can extract more diversity of compounds, which can explain the higher extraction efficiency. This result may be related, in part, to the fact that water helps in the diffusion of extractable compounds through plant tissues [35]. Ultrasound extraction offers numerous advantages when compared to other conventional extraction techniques such as solid-liquid extraction. An improved efficiency, reduced extraction time, and low solvent consumption makes this technique one of the most used [47]. Although in the current study the same extraction time and quantity of solvent were used for the ultrasound and solid-liquid extraction, the first technique, in general, did not lead to a higher extraction efficiency.

For Soxhlet and microwave extraction techniques using water as a solvent, the extraction efficiency was 13.4% and 11.9%, respectively (Table 1).

Bearing in mind all the solvents, for olive leaves, the average extraction yield with solid-liquid extraction was 5.1% ± 2.1% and for ultrasound extraction was 5.0% ± 1.8% (*p* > 0.05). For mimosa leaves, the values were 6.5% ± 1.7% and 4.4% ± 1.4% for solid-liquid and ultrasound extractions, respectively (*p* > 0.05). Considering all the extraction techniques used in this study, Soxhlet and microwave (using water as extraction solvent), provided the more efficient extraction for both plants (Table 1). The differences between them were not statistically significant (*p* > 0.05). Soxhlet has a great advantage in relation to the other methods used, as it is a very simple method that not require filtration after the extraction process. Furthermore, it allows for the extraction of more sample mass than most of the previous alternatives, such as microwave-assisted extraction. On the other hand, long extraction time is required and large amount of extractant is wasted [48]. In the study of Gallo et al. [49], the extraction yield of microwave method was also emphasized. These authors extracted phenolic compounds from different plants (*Cinnamomum zeylanicum*, *Coriandrum sativum*, *Cuminum cyminum,* and *Crocus sativus*), and the efficiency of extraction of bioactive compounds obtained with microwave extraction was in general about four times higher than that resulting from ultrasound extraction.

### 2.2. Antimicrobial Activity

The antimicrobial activity of olive and mimosa leaf crude extracts was evaluated against both Gram-positive (*Staphylococcus aureus*) and -negative (*Escherichia coli*) bacteria, in a first approach at no standardized concentrations, and then at a defined concentration (5 mg/mL; 0.05 mg/disc).

#### 2.2.1. Olive Leaf Extracts—Solid-Liquid and Ultrasound Extractions

The antimicrobial activity obtained from olive leaf extracts, extracted with solid-liquid and ultrasound methods against *S. aureus* and *E. coli*, are summarized in Table 2. The concentrations of the plant extracts for different extraction solvents were not standardized and depended of the volume of dimethyl sulfoxide (DMSO) used to dissolve the dry extracts.

Regarding the solid-liquid extraction, the concentration of the extracts ranged from 48.9 mg/mL (with hexane) to 254.1 mg/mL (with acetone). Antimicrobial activity was found for almost all solvents, except water against both *S. aureus* and *E. coli*, and dichloromethane against *E. coli* (Table 2). As in the present work, in the study of Korukluoglu et al. [33], the aqueous extract of olive leaves had no antibacterial effect against several Gram-positive and -negative bacteria, including those tested in this work. Nevertheless, some studies support that aqueous extracts of olive leaves have antimicrobial activity against pathogenic bacteria, including *S. aureus* (strains PTCC 1431, ATCC 6538, ATCC 43300, ATCC 25923, ESA 7, and MU40) and *E. coli* (strains PTCC 1399, CECT 101, ATCC 29998, ATCC 35218, and ATCC 25922) [25,50,51,52]. Therefore, perhaps if some conditions were different, for example, higher extraction time and/or temperature [53], the results could have been different for the aqueous extracts of the present study. Extracts of ethanol (25.3 mm) and acetone (22.7 mm) caused the highest inhibition halo (*p* < 0.05) against *S. aureus* and the differences between these two solvents were not significant (*p* > 0.05). According to Cowan [46], ethanol and acetone are highly efficient in the extraction of phenolic compounds. Indeed, this class of compounds are one of the main constituents of olive leaves [35]. Significant antimicrobial activity was also obtained with methanolic (16.7 mm) extracts followed by hexane (11.3 mm) and dichloromethane (8.7 mm) extracts. According to the results obtained by Mehmood et al. [39], compounds with antimicrobial activity can be extracted in ethanol and methanol. Also, they found that the great antimicrobial potential of methanolic extracts is associated with their high polarity, which enabled the extraction of all the phenolic compounds. Debib et al. [54], identified the presence of iridoids, flavonoids, and tannins in *O. europaea* methanolic and aqueous extracts (by solid-liquid extraction), which can be, in part, related with the antimicrobial properties observed against several bacterial (*S. aureus*-clinical isolate, *Bacillus subtilis* subsp. spizizenii-ATCC 6633), *E. coli*-ATCC 25922, *Enterobacter cloacae-*ATCC 13047, *Salmonella enterica* subsp. heindelberg-ATCC 8326, and *Klebsiella pneumonia*-clinical isolate) and fungal (*Candida albicans*) human pathogens.

Concerning the antimicrobial activity of the different extracts obtained against *E. coli,* no significant differences were observed between methanol (16.7 mm), ethanol (16.7 mm), acetone (14.7 mm), and hexane (12.0 mm) (*p* > 0.05). Comparing the antimicrobial activity on both bacteria tested, it was possible to observe that *S. aureus* (Gram-positive) was more susceptible than *E. coli* (Gram-negative) to olive leaf extracts obtained from solid-liquid extraction technique with ethanol, acetone, and dichloromethane as extraction solvent (*p* < 0.05). For methanol and hexane extracts, there was no significant differences in the effects caused by the plant extracts on both Gram-positive and Gram-negative bacteria (*p* > 0.05). Other works support the results obtained in the present study, where it has also been verified that Gram-positive bacteria are more susceptible to the olive leaf extracts tested (ethanolic and methanolic) compared with Gram-negative bacteria [39,55]. The main explanation for the higher or lower inhibition halos can be attributed to the differences in the structure of the cell envelope between Gram-positive and -negative bacteria. Regarding the Gram-positive bacteria, it is possible that the greater susceptibility observed in the olive leaf extracts may be associated with some specificity of one or more of their components on the bacterial cell wall [20]. The presence of an outer membrane in Gram-negative bacteria acts as a powerful barrier, restricting the diffusion of compounds through its lipopolysaccharide layer [56].

For ultrasound extraction, the concentrations of extracts ranged between 59.6 mg/mL (with methanol) and 239.3 mg/mL (with acetone) (Table 2). Ethanol was the extraction solvent that showed the best inhibitory activity against both bacteria tested (*S. aureus*—27.3 mm; *E. coli*—16.7 mm) (*p* < 0.05). Once again, for ethanolic extracts, *S. aureus* was the most susceptible bacterium (*p* < 0.05). Ethanol, with its ability to extract phenolic compounds [46], seems to be the best solvent to extract compounds with antibacterial activity from olive leaves using ultrasound extraction. Interesting inhibitory activity was also found with water (*S. aureus*—11.3 mm; *E. coli*—9.3 mm), methanol (*S. aureus*—18.7 mm; *E. coli*—16.7 mm), acetone (*S. aureus*—18.7 mm; *E. coli*—14.0 mm), and hexane (*S. aureus*—15.0 mm; *E. coli*—11.3 mm) extracts. However, for these extracts, no significant differences were observed among both bacteria (*p* > 0.05). No antimicrobial activity was found with dichloromethane extracts against *S. aureus* and *E. coli* (*p* < 0.05).

In order to obtain more consistent results on the relation between the extraction solvent used and antimicrobial effects, in a second approach the concentrations of the extracts were standardized and tested at 5 mg/mL (Table 3). Concerning solid-liquid extraction, the results were revealed as being very different–except for ethanol extracts, all the other extracts had no antimicrobial activity. Although, some activity was maintained for ethanolic extracts, a decrease in the inhibition halo of about 58% against *S. aureus* (*p* < 0.05) and 52% against *E. coli* (*p* < 0.05) was verified. However, at this concentration, no differences were found with ethanolic extracts among Gram-positive and -negative bacteria (*p* > 0.05). These results could indicate that it is necessary a concentration relatively high of extracts from olive leaves to have antimicrobial activity.

For ultrasound extraction, a standardized concentration of the extracts (5 mg/mL) was also applied and it was verified that methanol, dichloromethane, and hexane had no antimicrobial activity against *S. aureus* (Table 3). Inhibition halos of 10.7 mm was obtained with ethanol, and of 9.3 mm were obtained with both water and acetone extracts. For *E. coli*, only ethanol (10.7 mm) and acetone (10.7 mm) demonstrated inhibitory activity (*p* > 0.05). Compared to higher concentrations (no standardized concentrations), in general the inhibitory activity of the extracts decreased significantly (*p* < 0.05).

#### 2.2.2. Mimosa Leaf Extracts–Solid-Liquid and Ultrasound Extractions

The antimicrobial activity of mimosa leaf extract from solid-liquid and ultrasound extractions is shown in Table 2. Concerning solid-liquid extraction, the concentrations of the extracts ranged between 100.2 mg/mL (with dichloromethane) and 253.3 mg/mL (with ethanol). For *S. aureus*, the highest antimicrobial activity was exhibited with ethanolic extracts (28.0 mm), followed by methanol (16.7 mm), acetone (12.0 mm), and hexane (11.3 mm) (*p* < 0.05), whereas for *E. coli*, the best activity was achieved with methanol (16.7 mm), followed by ethanol (13.3 mm) and acetone/hexane (10.0 mm) (*p* < 0.05). Water and dichloromethane extracts did not show inhibitory activity against both bacteria.

For ultrasound extraction, the concentration of extracts ranged between 19.0 mg/mL (with hexane) and 181.5 0 mg/mL (with methanol) (Table 2). Ethanol was demonstrated as being the best extraction solvent on *S. aureus*, with an inhibitory halo of 25.3 mm. For this bacterium, inhibitory activity was also found with water (10.0 mm), methanol (9.3 mm), acetone (15.0 mm), and hexane (12.0 mm) extracts. No activity was found with dichloromethane extract against both *S. aureus* and *E. coli* and with water extract against *E. coli*. Methanol, ethanol, and acetone demonstrated similar activity against *E. coli*, with inhibition halos of 16.7 mm. Inhibitory activity was also observed with hexane (12.0 mm) extract.

Considering mimosa leaf extracts at 5 mg/mL obtained with solid-liquid extraction, only ethanol demonstrated antimicrobial activity against *S. aureus* (10.0 mm) and *E. coli* (9.3 mm) (Table 3). For the remaining extraction solvents, no antimicrobial activity was found. The same behavior was observed with ultrasound extraction for *E. coli* (ethanol—9.7 mm). For *S. aureus*, in addition to ethanolic extract (10.0 mm), aqueous extract (10.0 mm) showed similar inhibitory activity (*p* > 0.05). Reinforcing the present results, the antimicrobial activity of alcoholic *Acacia* extracts was previously documented [29,57,58,59].

#### 2.2.3. Olive and Mimosa Leaf Extracts–Soxhlet and Microwave Extractions

The extractions with Soxhlet and microwave used water as solvent and the concentrations of extracts obtained ranged from 82.3 mg/mL to 123.5 mg/mL (Table 2). Moreover, a standardized concentration of 5 mg/mL was also tested (Table 3). For *S. aureus*, at higher concentrations, there were no significant differences in the antibacterial activity found with both plants and between the type of extraction (olive/Soxhlet—14.0 mm; olive/microwave—14.3 mm; mimosa/Soxhlet—14.7; mimosa/microwave—14.3 mm) (*p* > 0.05). For *E. coli*, no antimicrobial activity was observed with mimosa leaf extracts. However, olive leaves extracts displayed inhibitory halos of 11.0 mm and 11.7 mm for Soxhlet and microwave extractions, respectively. Korukluoglu and collaborators [33] investigated the relationship between the extraction solvent (water, acetone, diethyl ether, and ethyl alcohol) using Soxhlet and the antimicrobial efficiency of different *O. europaea* L. leaf extracts against 15 bacteria, including *S. aureus* and *E. coli*. They found that the type of solvent affected the distribution and the amount of phenolics in the extracts and thus the antimicrobial activity.

A decrease in the concentration to 5 mg/mL led the extracts losing some activity, which was more evident against *S. aureus* (olive/Soxhlet—11.0 mm; olive/microwave—14.7 mm; mimosa/Soxhlet—10.3; mimosa/microwave—9.3 mm). Korukluoglu et al. [33] also reported that the inhibitory effects of the olive extracts increased with increasing concentration.

At 5 mg/mL, the extracts obtained from mimosa leaves did not show activity against *E. coli*. In fact, *Acacia* extracts are described as being less active against Gram-negative bacteria [60,61]. As mentioned previously, one possible explanation of the lack of activity of these plant extracts may be the presence of the outer membrane in the Gram-negative *E. coli* bacterium and/or the fact that the extraction method, solvent, or conditions (e.g., extraction time) were not the most appropriate to extract bioactive compounds from mimosa leaves. Şahin et al. [53] studied the optimum extraction conditions of olive leaf extracts using ultrasound-assisted as extraction method and ethanol as extraction solvent and demonstrated that extraction temperature, solvent concentration, and time are important variables.

When comparing the plant samples extracted with water for all the techniques, it was possible to verify that there was a better antimicrobial activity of the extracts from Soxhlet and microwave techniques in relation to those from solid-liquid and ultrasound. This confirms the advantages of Soxhlet and microwave methods to extract compounds with antimicrobial properties. This was also evident in other studies with different plants [62]. For instance, Gallo et al. [49] showed that in general the efficiency of microwave extraction to obtain bioactive compounds (in terms of total polyphenols and antioxidant capacity) from *Cinnamomum zeylanicum*, *Cuminum cyminum*, and *Crocus sativus* was about three, four, and six times higher, respectively, than ultrasound.

### 2.3. Antioxidant Capacity of Olive and Mimosa Leaves

In order to evaluate the antioxidant capacity of olive and mimosa leaves extracts, two techniques were applied, DPPH (2,2-diphenyl-1-picrylhydrazyl) and ABTS (2,2-azinobis (3-ethyl-benzothiazoline-6-sulfonic acid)). For both tests, the approach is based on electron transfer and involves reduction of a colored oxidant. The DPPH test is based on the reduction of purple DPPH to 1,1-diphenyl-2-picryl hydrazine [63]. Results of antioxidant capacity assessment are expressed in Trolox equivalent/g fresh mass, wherein Trolox equivalent is a synthetic vitamin E analogue [64]. ABTS test is based in the formation of ABTS blue/green, which can be reduced by antioxidants.

The results of antioxidant capacity for olive and mimosa extracts at 5 mg/mL obtained by the different methods (solid-liquid, ultrasound, Soxhlet, and microwave) with the selected solvents (water, methanol, ethanol, acetone, dichloromethane, and hexane) are presented in Table 4. Concerning olive leaves with solid-liquid extraction technique, acetonic extract showed the highest antioxidant potential by both DPPH and ABTS (*p* < 0.05). For ultrasound extraction, the extract obtained with acetone also demonstrated the best antioxidant capacity by ABTS, whereas by DPPH was the aqueous extract (*p* < 0.05). For both these extraction techniques (solid-liquid and ultrasound), methanolic and ethanolic extracts were those with lower antioxidant power based on DPPH (solid-liquid: methanol—166.5 Trolox equivalents (TE)/g fresh mass, ethanol—402.9 TE/g fresh mass; ultrasound: methanol—107.3 TE/g fresh mass, ethanol—372.3 TE/g fresh mass) and ABTS (solid-liquid: methanol—46.1 TE/g fresh mass, ethanol—210.4 TE/g fresh mass; ultrasound: methanol—184.3 TE/g fresh mass, ethanol—208.6 TE/g fresh mass) methods (*p* < 0.05). There are no doubts that the antioxidant capacity of plant extracts is related to extraction solvent used. For instance, polar solvents are most frequently employed for the recovery of polyphenols, which are one of the main groups of compounds responsible for antioxidant capacity [65]. In fact, ethanol, acetone, ethyl acetate, methanol, and aqueous mixtures of these solvents have been widely used on the extraction of compounds from plants and plant-based foods with antioxidant properties [65,66]. Thus, methanol and ethanol were not expected to provide extracts with lower antioxidant capacity. In fact, olive leaves’ methanolic extracts are usually rich in secoiridoids (e.g., oleuropein, ligostroside, dimethyloleuropein, and oleoside), flavonoids (e.g., apigenin, kaempferol, and luteolin), and phenolic compounds (e.g., caffeic acid, tyrosol, and hydroxytyrosol) that are associated with the antioxidant capacity [54].

Regarding the aqueous extracts, the best antioxidant potential was obtained with the microwave technique by both DPPH and ABTS methods. By the DPPH method, it was not observed significant differences between the extracts obtained from different types of extraction techniques (*p* > 0.05). The same did not happened by the ABTS method, as the solid-liquid extraction proved to be less efficient in extracting antioxidant compounds (*p* < 0.05).

In relation to mimosa leaves, with solid-liquid and ultrasound extraction techniques, acetonic (solid-liquid: 782.9 TE/g fresh mass; ultrasound: 770.1 TE/g fresh mass), aqueous (solid-liquid: 765.8 TE/g fresh mass; ultrasound: 756.5 TE/g fresh mass), and methanolic (739.8 TE/g fresh mass; ultrasound: 730.8 TE/g fresh mass) extracts proved to be those with higher antioxidant capacity on the basis of the DPPH method (*p* < 0.05), which may have been due to the presence of phenolic compounds.

Considering all extraction techniques using water as solvent, the highest values of antioxidant capacity by DPPH were found for solid-liquid, ultrasound, and Soxhlet extraction (*p* < 0.05). By the ABTS method, acetone was also one of the best solvents extracting antioxidant compounds with both solid-liquid (607.9 TE/g fresh mass) and ultrasound (607.9 TE/g fresh mass) extraction techniques. Moreover, dichloromethane (539.2 TE/g fresh mass) and ethanol (534.2 TE/g fresh mass) joined acetone as the best solvents in solid-liquid and ultrasound extractions, respectively (*p* < 0.05). No significant differences were found between the different types of extraction using water as solvent (*p* > 0.05). Sowndhararajan et al. [67] found that acetone extracts from barks of different species of *Acacia* (*A. leucophloea*, *A. ferruginea*, and *A. pennata*), including *A. dealbata,* had high antioxidant capacity (DPPH, ABTS, and OH; Ferric-reducing/antioxidant power; metal chelation; phosphomolybdenum reduction; and peroxidation inhibition methods). In the study of Luis et al. [8], high concentration of phenols was obtained from hydroalcoholic extract of *A. dealbata* (mimosa).

As mentioned above, the magnitude of the antioxidant capacity depends largely on the extraction solvent used [53,68]. For both olive and mimosa leaves, acetone was the one that allowed for the production of extracts with greater antioxidant potential, as stated by both methods (DPPH and ABTS). This likely happened due to its ability to extract phenolic compounds. In fact, acetone is very effective in extracting antioxidants such as phenolics, being one of the main solvents used to extract this class of compounds from plants [69]. Acetone has the ability to dissolve hydrophilic and lipophilic compounds [70]. Moreover, some characteristics of acetone such as volatility, miscibility with polar and non-polar solvents, and moderately low toxicity to some microorganisms (e.g., *S. aureus* and *Pseudomonas aeruginosa*), makes it a very useful extraction solvent [70].

Some studies have already reported the antioxidant capacity of olive [17,18,22] and mimosa leaf [8,71] extracts. The presence of oleuropein and other phenolic compounds (particularly containing hydroxyl groups), is an important factor for antioxidant capacity of olive leaf extracts [35,36,53]. Debib et al. [54] determined the total phenolic (from 3.64 to 21.47 mg gallic acid equivalents (GAE)/g dried matter) and flavonoid (from 3.33 to 17.64 mg catechin equivalents (CE)/g dried matter) contents of olive leaf extracts, using the solid-liquid extraction technique and three different extraction solvents (water, methanol (80%), and petroleum ether), and analyzed its correlation with the antioxidant capacity (as well as with the antimicrobial activity). The results indicated that the phenolic compounds appeared to be mainly responsible for the effects observed. In Abaza et al. [68], olive leaf extracts obtained using the solid–liquid extraction method and the four following extraction solvents—water, methanol (80%), ethanol (70%), and acetone (80%), presented a total phenolic content between 16.52 to 24.93 GAE/g dried matter and total flavonoid contents between 6.23 to 21.47 mg CE dried matter. These authors also verified that a methanol/water mixture was the best solvent to attain extracts rich in flavonoids and with considerable antioxidant properties. Lins et al. [22] conducted a study in order to evaluate the antioxidant potential of olive leaf extracts resulting from the Soxhlet extraction method and a mixture of methanol and water (ratio 80:20, v/v), as extraction solvent, and found total phenolic and flavonoid contents of 131.7 mg GAE/g dried weight and 19.4 mg quercetin equivalents (QE)/g dried weight, respectively. Recently, other studies on the characterization of the total phenolic content and antioxidant/composition profile were performed with extracts of *O. europaea* from Moroccan [72] and Italian [73] cultivars, using solid liquid extraction method and a solution of ethanol/water (80% and 60%, respectively) as solvent of extraction. Regarding the total phenolics, a range of values between 11 g/kg dried weight and 44 g/kg dried weight (Moroccan cultivars), and between 11.39 and 48.62 g GAE/kg dry weight (Italian cultivars), were obtained. The composition of the extracts consist predominantly of compounds belonging to the secoiridoids and flavonoids classes [72,73]. In this way, the amount of total phenolic compounds and the phytochemical profile are also dependent not only on the extraction method/solvent but also on the agronomical and environmental conditions and on the olive cultivars/varieties analyzed. These aspects were considered as being responsible for the variability on the content of total phenolics between different studies. Obviously, higher amounts of total phenolics is associated to a higher antioxidant capacity [72,73].

In *Acacia* species, the antioxidant properties can be attributed to the presence of phenolics and flavonoids [67]. Phenolic compounds have antioxidant activity mainly due to their redox properties, which allows them to act as reducing agents, hydrogen donors, and singlet oxygen quenchers [49,54]. In order to establish a possible relationship between the antioxidant capacity and the composition of the ethanolic, methanolic, acetonic, and hydroalcoholic crude extracts of *A. dealbata* obtained from different aerial plant parts including leaves using Soxhlet apparatus, the contents of total phenolics, flavonoids, and alkaloids was estimated by Luís et al. [8]. These authors found that the highest values of total phenolic were obtained with hydroalcoholic extracts (290.65 mg GAE/g dried mass) and the lowest with acetonic extracts (203.10 mg GAE/g dried mass). Total flavonoids and alkaloids ranged from 13.46 (hydroalcoholic extracts) to 21.70 (acetonic extract) mg QE/g dried mass and 5.81 (hydroalcoholic extracts) to 19.46 (acetonic extract) mg of pilocarpine nitrate equivalents (PNE)/g of dry mass), respectively. Moreover, they verified that uppermost antioxidant activity was displayed by the extracts with the highest total phenolic concentration. The presence of the following specific compounds: chlorogenic acid, syringic acid, p-coumaric acid, ferulic acid, and ellagic acid was identified in all of the four extracts. Quercetin was found in three of the extracts (except in acetonic extracts) and caffeic acid occurred only in the hydroalcoholic extracts.

Martysiak-Żurowska et al. [64] carried out a comparative study on the assessment of the total antioxidant capacity of human milk by the ABTS and DPPH methods, and concluded that the latter demonstrated lower sensitivity. These observations were probably due to limitations of the DPPH method that include restricted reactivity towards antioxidants and solubility in aqueous and organic solvents [74]. Instead of this, in the present study, the DPPH assay led, in most of the cases, to the highest values. For this reason, the DPPH method was considered a more useful method in the assessment of antioxidant capacity of olive and mimosa leaf extracts. Despite the differences observed between the values obtained by the DPPH and ABTS methods, for most of the cases they ranked the extracts similarly. In fact, both methods displayed that acetone extracts were the most effective free radical scavenger. This evidence is actually more relevant than knowing the precise chemical reactivity of each sample [75]. Comparing both plants used in the current study, differences were not observed between the antioxidant activities for the extracts with higher antioxidant properties (extracts of acetone) (*P* > 0.05). This proposes that olive and mimosa leaf extracts the have identical ability to scavenge free radicals.

## 3. Materials and Methods

### 3.1. Plant Origin and Sample Collection

The plants selected for this study were *O. europaea* and *A. dealbata*, collected from the area of Braga (Braga, Portugal). Fresh leaves were collected in February 2013 and immediately processed.

### 3.2. Preparation of Leaf Extracts

Four extraction methods (solid-liquid, ultrasound, Soxhlet, and microwave) and six extraction solvents (water, methanol, ethanol, acetone, dichloromethane, and hexane) (Sigma-Aldrich, Lisboa, Portugal) were used to prepare extracts from both plants. For every extraction method, fresh leaves washed with distilled water were cut in small pieces, approximately 5 mm in length and 4 mm in width, and freeze-dried before extraction. Five grams of leaves (wet weight) was used in each extraction procedure tested and the obtained extracts were evaporated in a rotary evaporator (Büchi Rotovapor R-114; Büchi Labortechnik AG, Flawil, Switzerland), except the water extracts, which were lyophilized. Dry extracts were then dissolved in dimethyl sulfoxide (DMSO; Sigma-Aldrich, Lisboa, Portugal) or methanol (Sigma-Aldrich, Portugal), depending on the test performed (antimicrobial or antioxidant capacity).

#### 3.2.1. Solid-Liquid Extraction

Leaves were placed in flasks with 50 mL of extraction solvent for 1 h, under magnetic shaking (Velp Scientific, Usmate, Italy) and maintained at 20 °C, using a Lovibond thermostatic cabinet (CABINET, Dortmund, Germany), according to the method suggested by Tomsone et al. [76].

#### 3.2.2. Ultrasound Extraction

The leaves were placed in a lidded flask with 50 mL of extraction solvent, and then put in a 420 W ultrasound bath (J.P. Selecta, Barcelona, Spain) at room temperature for 1 h [77].

#### 3.2.3. Soxhlet Extraction

Leaves were placed on a filter paper thimble (Whatman, Sigma-Aldrich, Portugal) inside of the main chamber of the Soxhlet (P Selecta, Barcelona) and 300 mL of water (extraction solvent) was placed on the top of the Soxhlet apparatus. A heating mantle was used to reflux the extraction solvent for 16 h, and after this time, the extract solution was allowed to cool to room temperature.

#### 3.2.4. Microwave-Assisted Extraction

Microwave-assisted extractions were performed according to Vera et al. [78]. Briefly, a modified version of the domestic oven (Electric Co. WP700P17-3; 2450 MHz, Harbin, China), with 1200 W input power and 2450 MHz frequency, was used. For this, a round bottom flask (100 mL of capacity) was placed inside the microwave and connected to a condenser. The flask contain leaves and 50 mL of water (extraction solvent), as well as some glass beads to avoid the overheating. A thermostatic bath (Thermo Fisher Scientific, Winsford, United Kingdom) was used to cool down the water (5 °C, used as cooling liquid). In order to verify if occur some leaks during the process, all experiments were conducted in a fume hood with a microwave radiation detector (MSM128, Meet Int., Hong Kong). The microwave was operated at a nominative output power of 397 W that is equivalent to an effective output power of 162 W [78], during 5 min [77].

### 3.3. Prevention of Bacterial Adhesion and Biofilm Formation

The extraction efficiency of each method was calculated by Equation (1):

(1) $$Extraction\;efficiency\;\left(\%\right)=\frac{m_{e}}{m_{l}}\times 100,$$

in which m_e_ is the mass of the extract obtained and m_l_ is the initial mass of the leaves, before extraction (wet weight).

### 3.4. Bacterial Strains and Growth Conditions

The bacteria used in this study were obtained from the Spanish Type Culture Collection (CECT): *Escherichia coli* CECT 434 and *Staphylococcus aureus* CECT 976. Before each experiment, both bacteria were cultured overnight (≈16 h) in flasks containing Mueller-Hinton broth (MHB; Merck, Darmstadt, Germany), at 37 °C under agitation at 150 rpm (Orbital IKA KS 130 basic, Staufen, Germany).

### 3.5. Antibacterial Activity Assessment

For the evaluation of the antibacterial activity of the olive and mimosa leaf extracts, a modification of the disc diffusion method originally described by Bauer et al. [79] was used. From an overnight culture grown on solid medium, a bacterial suspension of 0.5 McFarland standards (Spectrometer VWR V-1200) was prepared in sterile saline solution (NaCl 0.85% (w/v); Merck, Darmstadt, Germany). Then, Mueller-Hinton agar (MHA; Merck, Germany) plates were seeded with the respective bacterial suspensions and sterile blank discs (6 mm of diameter) (Oxoid, Madrid, Spain) impregnated with 10 µL of each extract placed on the top. Discs impregnated with DMSO were used as negative control. Lastly, the prepared plates were incubated at 37 °C for 24 h and the diameter (in mm) of the inhibitory zones around the discs was recorded [80].

### 3.6. Antioxidant Capacity Assessment

The antioxidant capacity of both *O. europaea* and *A. dealbata* leaf extracts was estimated by the evaluation of the ABTS (2,2-azinobis (3-ethyl-benzothiazoline-6-sulfonic acid)) and DPPH (2,2-diphenyl-1-picrylhydrazyl) radical scavenging activities, according to the methods proposed by Leong et al. [80] and Gil et al. [81], respectively.

Stock solutions of ABTS (Sigma-Aldrich, Portugal; 7.4 mM) and potassium persulfate (Sigma-Aldrich, Portugal; 2.6 mM) were prepared, mixed in equal quantities, and allowed to react for 12 h at room temperature in the dark. One milliliter of the resulting solution was diluted with 60 mL of methanol to obtain an absorbance value of 1.10 ± 0.02 at 734 nm. Then, the leaves extracts were mixed with the prepared solution (1:20 v/v) and allowed to react for 2 h in the dark. After the contact period, the absorbance was measured at 734 nm. A standard curve was performed using a reference antioxidant: 6-hydroxy-2,5,7,8-tetra-methylchroman-2-carboxylic acid (Trolox; Sigma-Aldrich, Portugal). The standard curve generated was linear between 25 and 800 µM Trolox. The results were expressed in µM Trolox equivalents (TE)/g fresh mass [82].

A stock solution of DPPH (Sigma-Aldrich, Portugal) (0.024 mg/mL) was prepared in methanol and then a portion of that (10 mL) was mixed with methanol (45 mL) in order to obtain an absorbance of 1.10 ± 0.02 at 515 nm. Then, 150 µL of each obtained extract was mixed with 2850 µL of the working solution and kept in the dark for 24 h. The absorbance was measured at 515 nm [82]. The standard curve generated was linear between 25 and 800 µM Trolox and the results expressed in µM TE/g fresh mass.

### 3.7. Statistical Analysis

The experiments were performed in triplicate with a minimum of three repeats. Data were analyzed using the one-way ANOVA test of the statistical program SPSS 21.0 (Statistical Package for the Social Sciences). Means were compared using Tukey–Kramer test. Significance level for the difference was set at *p* < 0.05.

## 4. Conclusions

This work allowed for the conclusion that extracts of *O. europaea* and *A. dealbata* leaves have antimicrobial and antioxidant activities. Methanol/acetone (olive leaf extracts) and water (mimosa leaf extracts) proved to be the solvents that provided higher extraction efficiencies for the solid-liquid and ultrasound extraction methods, respectively. Soxhlet and microwave (using water as extraction solvent) were the methods that were able to achieve the best extraction efficiency for both plant leaves. For solid-liquid and ultrasound extraction methods, ethanol seemed to be the best solvent used to obtain extracts rich in compounds with antimicrobial activity. Dichloromethane extracts were rarely active against the bacteria tested. Soxhlet and microwave extractions were the best techniques to extract compounds with antimicrobial activity, and *S. aureus* was more susceptible than *E. coli*.

In terms of antioxidant capacity, acetone was the best extraction solvent. No method of extraction excelled in the extraction of antioxidant compounds, and it largely depended on the extraction solvent used. The DPPH method provided the highest values for most of the cases. However, both DPPH and ABTS methods provided comparable conclusions in terms of antioxidant potential.

Overall, the results of the present study contributed to ascertaining which extraction method and solvent may be the most suitable for obtaining bioactive extracts from the selected plants. Moreover, it was highlighted that olive and mimosa leaf extracts can be considered as a potential source of natural antimicrobials and antioxidants. This is of valuable importance, as health hazards are frequently associated with the use of chemical antimicrobial agents or food preservatives.

## Figures and Tables

**Table 1:** Extraction efficiency (%) per extraction technique and solvent used with both olive and mimosa leaves.

Plants	Olive				Mimosa			
	Extraction Technique				Extraction Technique			
Solvent	Solid-liquid	Ultrasound	Soxhlet	Microwave	Solid-liquid	Ultrasound	Soxhlet	Microwave
Water	5.7 ± 0.9 ^c^	6.2 ± 0.9 ^c^	12 ± 1.2 ^d^	11 ± 0.8 ^d^	8.1 ± 0.8 ^a^	6.4 ± 1.2 ^a^	13 ± 1.4 ^b^	12 ± 1.8 ^b^
Methanol	7.2 ± 0.3 ^c^	6.0 ± 1.1 ^c^	-	-	7.9 ± 0.4 ^a^	5.7 ± 1.1 ^a^	-	-
Ethanol	4.1 ± 1.3 ^c^	4.6 ± 0.7 ^c^	-	-	6.0 ± 2.2 ^a^	4.2 ± 1.3 ^a^	-	-
Acetone	6.8 ± 1.1 ^c^	7.4 ± 0.3 ^c^	-	-	5.1 ± 0.8 ^a^	2.8 ± 0.0 ^a^	-	-
Dichloromethane	6.2 ± 1.6 ^c^	2.8 ± 0.3 ^b^	-	-	4.1 ± 1.5 ^a^	3.8 ± 2.1 ^a^	-	-
Hexane	0.9 ± 0.1 ^a^	3.2 ± 1.3 ^b^	-	-	7.8 ± 2.1 ^a^	3.7 ± 0.1 ^a^	-	-

(-)—not evaluated. For each plant and extraction method/solvent, different lower case letters were assigned in alphabetic order from the lowest to the highest value.

**Table 2:** Antibacterial activity of olive and mimosa leaf crude extracts at different concentrations obtained with the different extraction techniques and the selected solvents against *Staphylococcus aureus* and *Escherichia coli* (expressed in diameter of inhibition zone (mm)).

Plants	Solvent	Olive				Mimosa			
		Extraction Technique				Extraction Technique			
		Solid-Liquid	Ultrasound	Soxhlet	Microwave	Solid-Liquid	Ultrasound	Soxhlet	Microwave
***S. aureus***	Water	0.0 ± 0.0 ^a^	11.3 ± 1.2 ^c^	14.0 ± 0.8 ^d^	14.3 ± 1.2 ^d^	0.0 ± 0.0 ^a^	10.0 ± 1.8 ^b^	14.7 ± 0.5 ^c^	14.3 ± 0.5 ^c^
	Methanol	16.7 ± 1.2 ^e^	18.7 ± 1.2 ^e^	-	-	16.7 ± 1.2 ^c^	9.3 ± 0.6 ^b^	-	-
	Ethanol	25.3 ± 2.3 ^f^	27.3 ± 2.3 ^f^	-	-	28.0 ± 2.0 ^d^	25.3 ± 2.3 ^d^	-	-
	Acetone	22.7 ± 1.2 ^f^	18.7 ± 1.2 ^e^	-	-	12.0 ± 0.0 ^b^	15.0 ± 0.0 ^c^	-	-
	Dichloromethane	8.7 ± 0.6 ^b^	0.0 ± 0.0 ^a^	-	-	0.0 ± 0.0 ^a^	0.0 ± 0.0 ^a^	-	-
	Hexane	11.3 ± 1.2 ^c^	15.0 ± 1.4 ^d^	-	-	11.3 ± 1.2 ^b^	12.0 ± 0.0 ^b^	-	-
***E. coli***	Water	0.0 ± 0.0 ^a^	9.3 ± 0.6 ^b^	11.0 ± 0.0 ^c^	11.7 ± 0.5 ^c^	0.0 ± 0.0 ^a^	0.0 ± 0.0 ^a^	0.0 ± 0.0 ^a^	0.0 ± 0.0 ^a^
	Methanol	16.7 ± 1.2 ^d^	16.7 ± 1.2 ^d^	-	-	16.7 ± 1.2 ^d^	16.7 ± 1.2 ^d^	-	-
	Ethanol	16.7 ± 1.2 ^d^	16.7 ± 1.2 ^d^	-	-	13.3 ± 1.2 ^c^	16.7 ± 1.2 ^d^	-	-
	Acetone	14.7 ± 1.2 ^c^	14.0 ± 2.0 ^c^	-	-	10.0 ± 0.0 ^b^	16.7 ± 1.2 ^d^	-	-
	Dichloromethane	0.0 ± 0.0 ^a^	0.0 ± 0.0 ^a^	-	-	0.0 ± 0.0 ^a^	0.0 ± 0.0 ^a^	-	-
	Hexane	12.0 ± 2.0 ^c^	11.3 ± 1.2 ^c^	-	-	10.0 ± 0.0 ^b^	12.0 ± 0.0 ^c^	-	-

**Note:** Olive leaf extract concentration (mg/mL) for solid-liquid extraction—150.3 (water), 100.5 (methanol), 84.9 (ethanol), 254.1 (acetone), 253.2 (dichloromethane), and 48.9 (hexane). Olive leaf extract concentration (mg/mL) for ultrasound extraction—123.4 (water), 59.6 (methanol), 168.6 (ethanol), 239.3 (acetone), 93.4 (dichloromethane), and 161.3 (hexane). Olive leaf extract concentration (mg/mL) for Soxhlet extraction—89.3 (water). Olive leaf extract concentration (mg/mL) for microwave extraction—82.3 (water). Mimosa leaf extract concentration (mg/mL) for solid-liquid extraction—106.3 (water), 140.5 (methanol), 253.3 (ethanol), 190.4 (acetone), 100.2 (dichloromethane), and 145.7 (hexane). Mimosa leaf extract concentration (mg/mL) for ultrasound extraction—93.4 (water), 181.5 (methanol), 129.1 (ethanol), 27.5 (acetone), 24.4 (dichloromethane), and 19.0 (hexane). Mimosa leaf extract concentration (mg/mL) for Soxhlet extraction—123.5 (water). Mimosa leaf extract concentration (mg/mL) for microwave extraction—100.2 (water). (-)—not evaluated. For each bacteria, plant, and extraction method/solvent, different lower case letters were assigned in alphabetic order from the lowest to the highest value.

**Table 3:** Antibacterial activity of olive and mimosa leaf crude extracts at 5 mg/mL obtained with the different extraction techniques and the selected solvents against *S. aureus* and *E. coli* (expressed in diameter of inhibition zone (mm)).

Plants	Solvent	Olive				Mimosa			
		Extraction Technique				Extraction Technique			
		Solid-Liquid	Ultrasound	Soxhlet	Microwave	Solid-Liquid	Ultrasound	Soxhlet	Microwave
***S. aureus***	Water	0.0 ± 0.0 ^a^	9.3 ± 0.0 ^b^	11.0 ± 0.0 ^c^	10.8 ± 0.5 ^c^	0.0 ± 0.0 ^a^	10.0 ± 1.8 ^b^	10.3 ± 0.5 ^b^	9.3 ± 1.2 ^b^
	Methanol	0.0 ± 0.0 ^a^	0.0 ± 0.0 ^a^	-	-	0.0 ± 0.0 ^a^	0.0 ± 0.0 ^a^	-	-
	Ethanol	10.7 ± 0.6 ^c^	10.6 ± 0.4 ^c^	-	-	10.1 ± 0.8 ^b^	10.2 ± 1.0 ^b^	-	-
	Acetone	0.0 ± 0.0 ^a^	9.3 ± 0.6 ^b^	-	-	0.0 ± 0.0 ^a^	0.0 ± 0.0 ^a^	-	-
	Dichloromethane	0.0 ± 0.0 ^a^	0.0 ± 0.0 ^a^	-	-	0.0 ± 0.0 ^a^	0.0 ± 0.0 ^a^	-	-
	Hexane	0.0 ± 0.0 ^a^	0.0 ± 0.0 ^a^	-	-	0.0 ± 0.0 ^a^	0.0 ± 0.0 ^a^	-	-
***E. coli***	Water	0.0 ± 0.0 ^a^	0.0 ± 0.0 ^a^	10.0 ± 1.4 ^b^	9.7 ± 0.9 ^b^	0.0 ± 0.0 ^a^	0.0 ± 0.0 ^a^	0.0 ± 0.0 ^a^	0.0 ± 0.0 ^a^
	Methanol	0.0 ± 0.0 ^a^	0.0 ± 0.0 ^a^	-	-	0.0 ± 0.0 ^a^	0.0 ± 0.0 ^a^	-	-
	Ethanol	11.0 ± 0.2 ^b^	10.5 ± 0.8 ^b^	-	-	9.3 ± 0.8 ^b^	9.7 ± 0.6 ^b^	-	-
	Acetone	0.0 ± 0.0 ^a^	10.6 ± 0.6 ^b^	-	-	0.0 ± 0.0 ^a^	0.0 ± 0.0 ^a^	-	-
	Dichloromethane	0.0 ± 0.0 ^a^	0.0 ± 0.0 ^a^	-	-	0.0 ± 0.0 ^a^	0.0 ± 0.0 ^a^	-	-
	Hexane	0.0 ± 0.0 ^a^	0.0 ± 0.0 ^a^	-	-	0.0 ± 0.0 ^a^	0.0 ± 0.0 ^a^	-	-

(-)—not evaluated. For each bacteria, plant, and extraction method/solvent, different lower case letters were assigned in alphabetic order from the lowest to the highest value.

**Table 4:** Antioxidant capacity assessed by DPPH (2,2-diphenyl-1-picrylhydrazyl) and ABTS (2,2-azinobis (3-ethyl-benzothiazoline-6-sulfonic acid)) assays of olive and mimosa leaf crude extracts at 5 mg/mL obtained with the different extraction techniques and the selected solvents (expressed in TE/g fresh mass).

Plants	Solvent	Olive				Mimosa			
		Extraction Technique				Extraction Technique			
		Solid-Liquid	Ultrasound	Soxhlet	Microwave	Solid-Liquid	Ultrasound	Soxhlet	Microwave
**DPPH**	Water	687 ± 6.9	678 ± 4.0	739 ± 5.5	741 ± 10.8	766 ± 8.9	757 ± 4.2	699 ± 12	595 ± 2.1
	Methanol	167 ± 2.8	107 ± 7.9	-	-	740 ± 6.9	731 ± 4.8	-	-
	Ethanol	403 ± 5.7	372 ± 11	-	-	312 ± 8.8	477 ± 138	-	-
	Acetone	735 ± 7.9	539 ± 13	-	-	783 ± 4.7	770 ± 6.6	-	-
	Dichloromethane	561 ± 11	450 ± 3.4	-	-	226 ± 12	44.4 ± 4.5	-	-
	Hexane	323 ± 2.9	310 ± 6.9	-	-	84.4 ± 6.0	134 ± 8.7	-	-
**ABTS**	Water	415 ± 5.9	459 ± 12	450 ± 6.9	482 ± 9.7	357 ± 5.9	387 ± 5.5	339 ± 9.1	387 ± 9.8
	Methanol	46.1 ± 6.9	184 ± 12	-	-	475 ± 4.3	451 ± 6.6	-	-
	Ethanol	210 ± 6.8	209 ± 11	-	-	514 ± 6.9	534 ± 15	-	-
	Acetone	608 ± 4.4	719 ± 5.9	-	-	608 ± 5.9	608 ± 7.9	-	-
	Dichloromethane	518 ± 5.1	527 ± 4.9	-	-	539 ± 13	502 ± 11	-	-
	Hexane	383 ± 2.9	569 ± 11	-	-	393 ± 7.5	465 ± 5.9	-	-

(-)—not evaluated. For each bacteria, plant, and extraction method/solvent.
